# Does Epilepsy Have an Impact on Locus of Control?

**DOI:** 10.3389/fpsyg.2020.02251

**Published:** 2020-09-09

**Authors:** Peter Wolf, Katia Lin, Rüta Mameniškiené, Roger Walz

**Affiliations:** ^1^Danish Epilepsy Centre Filadelfia, Dianalund, Denmark; ^2^Programa de Pós-Graduação em Ciências Médicas, Universidade Federal de Santa Catarina, Florianópolis, Brazil; ^3^Department of Neurology and Neurosurgery, Center for Neurology, Vilnius University, Vilnius, Lithuania; ^4^Center for Applied Neuroscience, Hospital Universitário, Universidade Federal de Santa Catarina, Florianópolis, Brazil; ^5^Center for Epilepsy Surgery of Santa Catarina, Universidade Federal de Santa Catarina, Florianópolis, Brazil; ^6^Department of Internal Medicine, Neurology Service, Universidade Federal de Santa Catarina, Florianópolis, Brazil

**Keywords:** epilepsy stigma, seizure control, transcultural studies, epilepsy exclusion, quality of life, learned helplessness, chronic illness, religiosity and epilepsy

## Abstract

Many chronic diseases impair patients’ quality of life and may also affect their control perceptions. This could particularly happen for patients with epilepsy whose seizures often imply loss of control as a deeply disturbing experience. In 1980, a study on learned helplessness in epilepsy found a highly significant reduction of internal general locus of control (GLOC) and an increase of chance and powerful others health-related LOC (HLOC). In consequence, LOC became a frequent target of investigations relating to depression and anxiety, quality of life, coping, compliance, and other psychosocial aspects of epilepsy. Both GLOC and HLOC were investigated, and special groups like children, elderly, mentally handicapped persons, and those with psychogenic non-epileptic seizures were addressed. Most studies attempted to relate in-group differences of LOC to other parameters. Seizure-free patients were found to have a more internal HLOC, and patients with severe epilepsies have a more external HLOC. Patients with a high external HLOC seem to have more difficulties with coping and to be more anxious. Whereas external GLOC was correlated with learned helplessness, internal GLOC was associated with high self-efficacy and better life quality. An association of external LOC with depression seemed not to be a stable co-relation as clinical improvement following epilepsy surgery dissociated the two. A hypothesis was confirmed that the ability of some patients to counteract seizures at their onset, thus preserving control, was correlated with a higher internal HLOC. Some other theoretically well-founded hypotheses were not supported. Absolute figures as reported in several papers are of limited use because the only normative data for comparison come from a local sample of 1976 from Tennessee, whereas LOC scores may differ largely dependent on cultural and societal conditions. Very few controlled studies exist, and the early finding of a generally externalized LOC in epilepsy was confirmed only in one study performed in a South Indian community known for strong stigma against epilepsy. A recent transcultural investigation conducted in Brazil and Lithuania found no differences from healthy controls and between countries. It seems worthwhile to further investigate relations of LOC with epilepsy stigma.

## Introduction

Many chronic diseases have an impact on people’s quality of life (QOL) and may also affect their control perceptions. Since epileptic seizures often imply an objective loss of control, the assessment of control perceptions of epilepsy patients is relevant. To rate these, Rotter (1966) has developed the concept of internal versus external locus of control (LOC), and a 29-item scale to measure it. To review the literature studying LOC in epilepsy, we searched in PubMed for MeSH topics (Epilepsy) AND (Locus of control) and supplemented the retrieved manuscripts by all references that could be extracted from them.

Locus of control in epilepsy was first investigated by DeVellis et al. (1980) in the United States who, in a study on learned helplessness in 286 individuals with epilepsy, addressed the hypothesis that seizures, especially when they were frequent, severe, and difficult to predict and control, would result in reduced internal control over outcome and possibly increased depression. Both general LOC (GLOC) and health-related LOC (HLOC) were assessed. For HLOC, the Multidimensional Health Locus of Control scales (form A) of Wallston et al. (1978) (henceforth abbreviated as W78) were used that distinguish between internal, chance, and powerful others control. For GLOC, they applied scales that had been developed by Levenson (1973) as a “modification of Rotter’s (1966) Internal–External Locus of Control scale in order to measure more accurately expectancies of control as they relate to adjustment and clinical improvement.” The Levenson scales likewise distinguish internal, chance, and powerful others control. They had in fact served as a model for the development of the W78 scales. To reduce the test material, the Levenson powerful others scale was omitted in the DeVellis et al. (1980) study because it had been shown to be moderately correlated with the chance scale. All LOC findings in patients were compared with a normative sample of 115 chance individuals recruited in 1976 at Nashville Municipal Airport as reported by Wallston et al. (1978). The patients showed a significant reduction in internal GLOC and increase in chance HLOC, whereas internal and powerful others HLOC and chance GLOC did not differ significantly from the normative sample. The authors performed a hierarchical-regression analysis where they tested for (1) auras (i.e., initial seizure symptoms that are consciously perceived by the patients as a “warning” of the seizure), (2) believes to be able to avoid or terminate a seizure voluntarily, (3) relation of seizures to certain situations, (4) seizure severity and several other parameters. The regression analysis revealed that having auras and the belief of being able to prevent or stop a seizure were associated with increased internal GLOC and HLOC and decreased depression. Predictability of seizures was correlated with higher internal HLOC, whereas increased severity, early onset, and longer duration of epilepsy were associated with lower internal HLOC but increased chance and powerful others LOC, as well as increased depression.

With these findings, externalization of LOC in epilepsy was considered an established fact, and LOC instruments were in consequence often applied in epilepsy both in children and adults.

## Studies of Health-Related Locus of Control

Several studies on LOC in epilepsy focused on HLOC because they were particularly interested in the impact of control perceptions on disease-related parameters (Table 1).

**TABLE 1:** Literature review since 1980.

Authors, year	Country	Subjects (N)	LOC Instruments	Main findings
**Seminal study**				
DeVellis et al., 1980	United States	286 PWE	W78 Levenson scales for GLOC	• Patients had reduced internal GLOC and increased chance HLOC, whereas internal and powerful others HLOC, and chance GLOC did not differ from normative sample • Having auras and the belief of being able to prevent or stop a seizure were associated with increased internal GLOC and HLOC, and decreased depression
**Studies of Health LOC**				
Rosenbaum and Palmon, 1984	Israel	50 PWE	Rosenbaum’s Self-Control Schedule W78 (modified)	• HR patients had a more internal HLOC and a higher perception of seizure control • HR patients were less depressed and anxious. Their coping was more successful than in LR patients, but only as long as seizure frequency was low to moderate
Smith et al., 1991	United Kingdom	100 patients with medically refractory focal seizures	Rotter’s LOC?	• Seizure severity was the most significant predictor of LOC, anxiety and self-esteem
Chovaz et al., 1994	Canada	42 drug-resistant patients submitted to temporal lobectomy	W78	• Psychosocial outcome was better with good seizure control, whereas depression and lack of resourcefulness were correlated with poor psychosocial function • HLOC had no influence on psychosocial outcome
Krakow et al., 1999	Germany	40 patients with intractable epilepsy	IPC-questionnaire measuring generalized LOC	• The use of coping patterns, which were regarded as maladaptive, was correlated with distinct depression, a small degree of internal LOC beliefs and poor psychosocial adaption
Spector et al., 2001	United Kingdom	100 PWE (uncontrolled seizures)	W78	• No significant differences in the internal and chance scales whereas patients identified as low controllers believed more in control by powerful others • Higher internal and lower powerful others HLOC scores were associated with lower depression scores, and higher internal HLOC was associated with higher learned resourcefulness and higher self-esteem
Gramstad et al., 2001	Norway	101 PWE	W78	• The hypothesis that HLOC is of importance for the psychosocial functioning in patients with epilepsy was not supported by this study
Au et al., 2002	Hong Kong	67 with active epilepsy	W78	• Chance HLOC was the only one which correlated with health-related QOL • Health locus of control and the satisfaction with social support confirmed the importance of the influence of the subjective sense of mastery of condition on quality of life
Asadi-Pooya et al., 2007	United States	200 PWE (60 seizure-free)	W78 (form C)	• PWE had weak perceptions of internal and strong perceptions of external HLOC • Higher internal LOC was related with being seizure free. Patients with higher powerful others rating had increased anxiety levels, whereas no correlation with depression was found
Lohse et al., 2015	Denmark	49 PWE	W78 (form C)	• Ability to react to an aura prior to a seizure relates to higher internal HLOC but not to levels of anxiety and depression
**Studies of General LOC**				
Hermann and Wyler, 1989	United States	37 PWE submitted to neurosurgical treatment	Rotter’s LOC	• Preoperatively there was a correlation between external LOC and depression. Postoperatively, depression improved associated with seizure freedom, LOC did not change
Hermann et al., 1990	United States	102 PWE (uncontrolled seizures)	Rotter’s LOC	• External LOC showed a significant relationship with the general health
Gehlert, 1994, 1996	United States	143 PWE	Rotter’s LOC W78	• Perceptions of control were conceptualized as learned helplessness for bad, but not for good, events. The hypothesis that individuals who continue to have seizures become more and more external in perceptions of control was not confirmed
Amir et al., 1999	Israel	89 PWE	Rotter’s LOC	• Ninety percent of the variance of the WHO-QOL was explained by a combination of disease severity, self-efficacy in epilepsy, social support, and locus of control
Gopinath et al., 2000	India	200 PWE	Rotter’s LOC (modified)	• Most patients had an external locus of control, which negatively influenced their compliance
Yeni et al., 2016	Turkey	70 PWE	Rotter’s LOC	• LOC correlated with anxiety and depression, but did not correlate with patients’ attitudes toward epilepsy, epilepsy knowledge, quality of life and stigma
Moritz et al., 2018	Brazil and Lithuania	186 PWE 189 healthy controls	Rotter’s LOC	• GLOC score did not differ between patients and controls nor between Brazilians and Lithuanians • Having auras, reacting to them and being able to avoid seizures had no effect on any GLOC score but on QOL and anxiety
**Children**				
Matthews and Barabas, 1986	United States	15 children with epilepsy 15 children with diabetes 15 healthy children	MMCPC	• Children with epilepsy displayed the greatest perception of an external source of control relative to other children (not significant)
**Elderly**				
McLaughlin et al., 2010	Australia	64 PWE ≥ 60 yo	Rotter’s LOC	• More external LOC, which did not predict impaired health-related quality of life
**Mentally handicapped persons**				
Espie et al., 1990	Scotland	65 PWE	Nowicki-Strickland LOC scale	• Mentally handicapped adults perceived external control over their environment in view of the lack of autonomy offered to them
**Psychogenic non-epileptic seizures**				
Moore et al., 1994	United Kingdom	19 PWE 19 PNES 19 healthy volunteers	Levenson scales	• No significant differences were found between the three groups regarding the scores for LOC
Stone et al., 2004	Sweden	20 PWE 20 PNES	Swedish 50-item self-report instrument for assessing LOC	• Patients with PNES had a more external locus of control
Strutt et al., 2011	United States	30 women with PNES 51 women with temporal lobe epilepsy	W78 (form C)	• PNES patients had a higher score on significant others HLOC, but no general differences in health-related LOC

*GLOC, general locus of control; HR, high-resourceful; LR, low-resourceful; MMCPC, Connell’s multidimensional measure of children’s perception of control; PNES, psychogenic non-epileptic seizures; PWE, persons with epilepsy; QOL, quality of life; W78, Wallston’s health-related locus of control scale (1978).*

Soon after the DeVellis et al. (1980) paper, Rosenbaum and Palmon (1984) looked into the influence of helplessness and resourcefulness on coping with epilepsy. Fifty patients with epilepsy of three grades of severity (according to the frequency of generalized tonic−clonic seizures) were divided into a high-resourceful (HR) and a low-resourceful (LR) group according to their scores on Rosenbaum’s Self-Control Schedule. This instrument was used as a measure for learned resourcefulness, i.e., individual tendencies to apply self-control methods to the solution of behavioral problems. State and trait anxiety and depression were assessed by established instruments, and HLOC was measured by an earlier (1976) version of W78, supplemented by a scale constructed *ad hoc* for perception of control of seizures. Coping was rated by the Acceptance of Disability scale developed by Linkowski. Half of the patients each belonged to the HR and LR groups. There was no control group. The authors found that HR patients were less depressed and anxious. Their coping was more successful than in LR patients, but only as long as seizure frequency was low to moderate. If it was high, there was no difference. HR patients had a more internal HLOC and a higher perception of seizure control. No independent correlation of HLOC and coping success was reported.

As part of the development of the Liverpool seizure severity scale, Smith et al. (1991) studied the psychosocial consequences of seizure frequency and severity, and external LOC was one of the investigated parameters. Due to a mistake in references, it is unfortunately not clear what scale was applied on the 100 patients with focal epilepsies who were included. Probably it was the Rotter (1966) scale. Seizure severity was found to be the most significant predictor of self-esteem (*p* = 0.005), LOC (*p* = 0.039), and anxiety (*p* = 0.048). Of note, the severity scale included some items examining how much control the patients had over their seizures. Seizure frequency had no influence on the variance of psychological factors.

Chovaz et al. (1994) investigated factors influencing the psychosocial function, measured by the Washington Psychosocial Seizure Inventory (WPSI, Dodrill et al., 1980) and a structured interview, after treatment of 42 drug-resistant patients by temporal lobectomy. Resourcefulness, depression, and LOC were assessed as variables of learned helplessness. Psychosocial outcome was better with good seizure control, whereas depression and lack of resourcefulness were correlated with poor psychosocial function. HLOC (W78) had no influence.

Coping was, again, the aspect that was addressed by Krakow et al. (1999). They applied a German version of W78 together with the Freiburg Questionnaire for Coping with Illness, which considers five dimensions of coping, von Zerssen’s scale for state depression, and the Social Interview Schedule for psychosocial adaptation. Forty patients with uncontrolled seizures were included. The authors were particularly interested in the relations of different coping strategies. They found that a high score of powerful others HLOC was correlated with religiousness/search for meaning, dissimulation/wishful thinking, and depressive coping, but also with an integrating scale of ineffective coping. Chance HLOC beliefs were correlated with unfavorable social management, and patients with a low internal LOC tended to have maladaptive coping patterns.

Spector et al. (2001) returned to the question of resourcefulness and divided their 100 patients with uncontrolled seizures into a high controller (HC) and a low controller (LC) group. This was unequivocally possible only in 79 subjects of whom 58 belonged to the HC, and 21 to the LC group. They wanted to replicate the findings of Rosenbaum and Palmon (1984) and, in addition, to clarify what strategies the patients applied to control seizures. Instruments applied were W78, the Hospital Anxiety and Depression Scale (HADS), and Rosenberg’s self-esteem scale. The control strategies were assessed in a semi-structured interview based on six questions referring to both facilitating and counteractive factors of seizures.

(1) Have you noticed any situations or states that will cause you to have more seizures?
(2) Have you ever got yourself into any of these states on purpose, knowing you will probably have a seizure?
(3) Have you ever encouraged a seizure to come (can you bring on a seizure)?
(4) Are there any situations or states in which you have fewer seizures?
(5) Do you sometimes make yourself have fewer seizures (e.g., by avoiding seizure precipitants)?
(6) Can you sometimes stop your seizures from happening?

Whereas affirmative answers to the first three (facilitator) questions were similar in the HC and LC groups, HC patients gave significantly more positive answers to questions 4–6 (counteractive). LC patients were more often women, were more likely to have focal seizures with impaired awareness, and significantly more often identified one or more seizure precipitants. Regarding HLOC, there were no significant differences in the internal and chance scales, whereas LC patients believed more in control by powerful others. Higher internal and lower powerful others HLOC scores were associated with lower depression scores, and higher internal HLOC was associated with higher learned resourcefulness and higher self-esteem. Higher age and longer duration of epilepsy were correlated with higher powerful others HLOC.

The authors had expected to find that “the occurrence of warnings would enable people to abort their seizures more readily and hence be associated with high perceived self-control. This prediction was not borne out by the present data.” However, near-reading the article does not show that this question was tested directly. The conclusion seems, rather, to have been indirect: affirmative responses to questions 4–6 “were the main behaviors contributing to the probability of being in the HC group” but HC was not related with higher internal or lower chance HLOC, only with lower significant others HLOC. The wording in the article is compatible with this but cannot be understood to mean that the prediction was refuted. In addition, the data did not allow telling if patients’ attempts at aborting seizures reduced their seizure frequency and whether this had an influence on their control perceptions.

Gramstad et al. (2001) in Norway investigated the hypothesis that negative and positive affectivity, self-efficacy, and HLOC are important for psychosocial adjustment in patients with epilepsy. They included 101 patients who were tested with W78 and the WPSI (Dodrill et al., 1980), an extensively and cross-culturally validated instrument to measure psychosocial adjustment in patients with epilepsy. It has seven distinct dimensions and one summary measure of overall social function. In addition, the positive and negative affect schedule (PANAS-X), and established scales for general and epilepsy-related self-efficacy were included. The Wallston scale has three dimensions (internal, chance, and powerful others) with six items each that apply a Likert scale of 1–6. Thus, every item has a possible range of 6–36. The scores found were (mean ± SD) 22.71 ± 5.48 for internal, 19.37 ± 6.2 for chance, and 21.06 ± 6.25 for powerful others. In comparison, the normative data for healthy adults of W78 are 25.37 ± 5.32 for internal, 16.23 ± 6.28 for chance, and 20.23 ± 5.49 for powerful others. All figures thus indicated externalized HLOC in the patients, but the standard deviations were large, and the study had no control group. Regarding intercorrelations between the measures studied, they were good for affects, self-efficacy, and clinical items of WPSI, whereas correlations with HLOC were low or insignificant. The authors concluded that “the hypothesis that HLOC is of importance for the psychosocial functioning in patients with epilepsy was not supported by this study.”

Au et al. (2002) reported on the QOL in Hong Kong Chinese adults with epilepsy. They investigated health-related QOL (HRQOL) with the QOLIE-89 and HLOC with W78. In addition, the HADS and a social support questionnaire were applied. Sixty-seven patients with active epilepsy were enrolled. The HLOC scores were 24.97 ± 4.79 for internal, 18.87 ± 6.48 for chance, and 25.63 ± 5.08 for powerful others. Of these, chance HLOC was the only one that correlated with HRQOL but only weakly and as long as the data were not controlled for the influences of mood.

Asadi-Pooya et al. (2007) investigated the relationship between HLOC and anxiety, depression, and seizure control in 200 patients of an outpatient clinic and an epilepsy monitoring unit at Thomas Jefferson University, Philadelphia (United States). Sixty patients were seizure-free, the others not. They applied the more recent form C of W78, which was developed to be adaptable to specific health conditions (Wallston et al., 1994), together with the HADS. HLOC scores were 19.6 ± 6.3 for internal, 17.7 ± 6.4 for chance, and 24.4 ± 5.4 for powerful others. There was no control group, and for this form, there are no normative data. Stepwise regression revealed some associations. A higher internal LOC was significantly related with being seizure–free, whereas the two other subscales were not associated with seizure control; patients with higher powerful others rating had increased anxiety levels, whereas no correlation with depression was found. The authors conclude that the powerful others LOC in these cases relates to a higher need for advice to deal with their anxiety: “Physicians should be aware of their strong role in determining their patients’ health-related beliefs and behaviors and be more solicitous of their thoughts and desires. Further studies are required to better clarify the significance of patient–physician communication in this regard and how HLC may be related to improving the management of patients with epilepsy.”

Lohse et al. (2015) tested the hypothesis that patients whose seizures start with an aura to which they can react in a meaningful way would experience less loss of control by seizures and have a more internal LOC. Of 98 eligible patients, 49 participated in the study. They submitted per mail an aura questionnaire, the HAD scale, and form C of Wallston’s HLOC scale. This was followed up by a semi-structured telephone interview. Twenty-eight reported auras to which they could react. The others had no auras or could not react. Aura experiences as such had no significant correlation with any HLOC scores, but patients who had auras and could react to them scored 22.0 (17.3–26.0) on internal HLOC, the others 14.0 (11.0–22.5, *p* = 0.017). They also scored lower on the chance and powerful others scale, but these differences were not significant. Both groups did not differ in the anxiety and depression measures, which were within the normal range. The hypothesis was, thus, confirmed.

To conclude, some studies confirmed low internal or high external LOC scores for patients with epilepsy compared with a normative sample, whereas no controlled studies exist. A positive correlation of auras and possibilities of counteracting seizures with internal LOC, as reported by DeVellis et al. (1980) was confirmed by one study, whereas another had an equivocal conclusion. More internal scores were found in seizure-free patients and those with high resources, whereas more severe seizures predicted external HLOC. HLOC was not correlated with psychosocial functions after neurosurgical treatment of epilepsy, and correlation with life quality was questionable. High external HLOC was correlated with ineffective and maladaptive coping, and increased anxiety in patients with a high powerful others HLOC was reported in one study but denied in another.

## Studies of General Locus of Control

Another series of studies investigated general LOC to find whether the frequent experience of loss of control by unexpected seizures would affect control perceptions not only relative to health but to life in general (Table 1).

Authors were often primarily interested in the correlation of within-group differences of LOC in epilepsy patients in relation with other psychosocial aspects, without reporting the patients’ LOC data.

Hermann and Wyler (1989) studied the relation between external LOC (using Rotter’s external–internal scale) and depression in 37 patients who underwent neurosurgical treatment for epilepsy. Preoperatively, there was a correlation between external LOC and depression. However, whereas depression was found significantly improved 6 months postoperatively in the 22 individuals who were rendered seizure-free, the LOC did not change, contrary to the authors’ expectation. LOC seemed, thus, to be much less dependent than depression upon the present state of health. They conclude that LOC is a learned phenomenon acquired over the entire lifetime. Patients had suffered from epilepsy in average for 18 years, and it was perhaps unreasonable to expect a change after only 6 months without seizures.

The same group (Hermann et al., 1990) assessed the psychiatric status of 102 patients with epilepsy using Goldberg’s General Health Questionnaire. They investigated the possible predictive value of many factors for psychopathology, and external LOC (measured with the 1966 Rotter internal–external scale) was one of seven identified variables found (*p* = 0.017). However, after stepwise multiple regression analysis, it was not retained as an independent variable.

Gehlert (1994, 1996) pointed out that externality of control and learned helplessness had become included in multietiologic, theory-based models of psychosocial problems in epilepsy, but these models had never been tested. The existing empirical studies had shown an association of externality of control and learned helplessness with epilepsy but not given insight into the nature of these associations. Her study therefore addressed the hypothesis that seizure-free individuals would have more internal control perceptions than those still having seizures. To take care of the time factor in the development of control perceptions either way, an “index of seizure control” was calculated as seizure-free years divided by present age minus age at epilepsy onset. It was a questionnaire study mailed to 782 patients, with 143 (22%) completed and returned. The instruments used were the Rotter scale for internal vs. external LOC, W78, and the Attributional Style Questionnaire of Peterson et al. (1982) for learned helplessness. The main hypothesis was not confirmed for either GLOC or HLOC, only attributions as helplessness for bad events were significantly reduced in relation to the index of seizure control. LOC and learned helplessness did not appear isomorphic.

In a different approach by Amir et al. (1999), GLOC was along with the Self-Efficacy Scale (SES), both in Hebrew adaptations, considered as dimensions of mastery that, together with social support, could mediate between disease severity (measured with the Liverpool Seizure Severity Scale) and QOL. Life quality of 89 patients with active epilepsy was determined with the WHO Quality of Life questionnaire (WHOQOL). “Ninety percent of the variance of the WHOQOL was explained by a combination of disease severity, self-efficacy in epilepsy, social support, and locus of control. Mastery was found to mediate the correlation between disease severity and QOL, and social support was found to act as a mediator between disease severity and mastery.” More internal LOC was correlated with higher self-efficacy and higher QOL.

Gopinath et al. (2000) in Kerala, South India, investigated 200 patients with epilepsy using the Rotter scale (1966) in a version standardized and validated for the local population. Their answers were categorized as internal, intermediate, or external using the 33rd and 67th percentile of a control group of 206 healthy adult volunteers. The I-E score (mean ± SD) of the patients was 0.355 ± 0.214 compared with 0.287 ± 0.204 in the control group (*p* < 0.001), and the majority of patients (45.2%) had a score above the 67th percentile.

These authors were interested in the relation of LOC, doctor–patient communication, and compliance with prescribed medications and advice about adequate behavior. For assessment of communication and compliance, they used a semi-structured interview following the consultation based on a self-developed questionnaire. There was a significant positive correlation between a good doctor–patient communication and good compliance. In bivariate analysis, non-compliance was correlated with more external LOC (*p* = 0.022), whereas in multivariate analysis, this trend did not reach statistical significance.

Yeni et al. (2016) studied the attitudes of 70 Turkish epilepsy patients toward epilepsy and found that these were primarily influenced by knowledge about epilepsy, stigma, depression, and related with QOL. GLOC was assessed with a validated Turkish version of the Rotter (1966) instrument where they had an average score of 10.5 ± 3.32 (no control group). LOC did not appear correlated with patients’ attitudes.

As a consequence of our earlier study (Lohse et al., 2015) that had shown that patients’ ability to react meaningfully to auras was correlated with a higher internal score on Wallston’s HLOC scale (form C), Moritz et al. (2018) wanted to know if the same was true for GLOC. The idea behind was that the repetitive experience to be able to avoid loss of control by seizures would provide a higher perception of self-control not just regarding their epilepsy but for their lives in general. A transcultural study was conducted comparing control perceptions in Brazil and Lithuania. The two countries belong both, in general terms, to the well-developed democratic Western societies but at the same time differ in other important respects: Brazil belongs to the “rising economies” of the Southern hemisphere, with strong traditional religious attitudes and a still only moderately developed middle class. Lithuania belongs to the Northern hemisphere, has a strongly secularized society and recently experienced the societal revolution of a move from the Soviet Union to the European Union.

Being aware of some methodological issues of LOC research, the authors decided to apply the original internal–external LOC scale of Rotter (1966) but included healthy controls for comparison. The scale consists of 29 questions where participants have to choose between two opposing statements representing an internal or external view.

The score is given as the sum of all answers indicating an external view. As six questions serve as distractors, the scale reaches from 0 (maximum internal LOC) to 23 (maximum external LOC). In addition, the HADS and QOLIE-31 were applied as the well-established instruments for anxiety, depression, and QOL in epilepsy. Religiosity was assessed with the Index of Core Spiritual Experiences-Revised (INSPIRIT-R). Data were collected in parallel in both countries separately using the same procedures. A total of 186 patients and 189 controls were recruited. 111 patients were enrolled in Lithuania and 75 in Brazil. The results were surprising: the GLOC score differed neither between patients (9.56 ± 3.46) and controls (8.96 ± 3.34) nor between Brazilians (9.08 ± 2.87) and Lithuanians (9.88 ± 3.79). Patients had a lower level of education and lower social status; they had a higher rate of unemployment and a higher score on the depression scale. Brazilians had a shorter education, were more religious, and scored higher for anxiety.

Having auras, reacting to them and being able to avoid seizures had no effect on any GLOC score, which makes sense as these were not deviant anyway. However, patients with auras had a lower life quality and a higher level of anxiety (perhaps because they are more aware of their seizures), but this difference disappeared when they had the experience of being able to actively avoid seizures.

To conclude, controlled studies confirmed high external GLOC of patients with epilepsy in South India in 2000 but not in Brazil and Lithuania in 2018. Apparent correlations of external LOC with increased psychopathology and non-compliance were not confirmed in multiple regression analysis, and there was no correlation of GLOC with patients’ attitudes about epilepsy. Whereas external GLOC was correlated with learned helplessness, internal GLOC was associated with high self-efficacy and better life quality. A correlation between external LOC and depression that is indicated in several studies disappeared after successful epilepsy surgery because depression improved rather rapidly, whereas external GLOC did not.

## Locus of Control in Special Groups

### Children

Matthews and Barabas (1986) in a much quoted study tested 15 children with epilepsy with Connell’s Multidimensional Measure of Children’s Perception of Control (MMCPC) and compared them with two age-matched groups of 15 each, children with diabetes and healthy controls. Additional instruments used were the Piers–Harris Self-Concept Scale, the Draw-a-Person test, and the Rochester Adaptive Behavior Inventory. They reported that “children with epilepsy invariably displayed the greatest perception of an external source of control relative to other children.” However, the figures show that, in the overall means, the figures of the children with epilepsy were only slightly above diabetes, and statistical difference was only found for “unknown source of control” between both chronically ill groups and the healthy controls, whereas the figures for powerful others were small and showed no statistical differences at all. Thus, the findings with this small sample may perhaps have been overinterpreted.

### Elderly

McLaughlin et al. (2010) studied the impact of epilepsy on the QOL of older people in Australia. Sixty-four community-dwelling individuals aged 60 or above were included. The HRQOL instrument QOLIE-31 was applied as well as the Composite International Diagnostic Interview (CIDI)-Auto, a computerized, structured interview, for the detection of depression and dysthymia. Seizures were categorized as “partial” versus “generalized” onset, with four categories of frequency. GLOC was measured with the scale of Rotter (1966) and gave a score of 13.28 ± 4.08. This might indicate more external LOC, but there was no control group. Contradictory to expectations, external HLOC did not predict impaired HRQOL.

### Mentally Handicapped Persons

Espie et al. (1990) investigated LOC in mentally handicapped persons in Scotland. Their prediction was that people with the double burden of seizures and mental handicap would display a higher amount of external LOC. They set out to investigate “the effects of polypharmacy and seizure frequency upon psychosocial behavior and examine locus of control orientation in a community sample of mentally handicapped adults with epilepsy.” Of the 65 subjects included, 21 had a high frequency of more than one seizure/month (HF), the others a lower frequency (LF). Psychosocial behavior was assessed with a psychosocial behavior scale (PBS) that had been previously developed by the authors. It indexes the frequency and intrusiveness of problematic behaviors. For LOC, the Nowicki and Strickland (1973) LOC scale for children (1973) was applied in a subgroup of patients forming three matched groups of nine individuals each for comparison: HF patients, LF patients, and controls with no history of epilepsy. The 40-item scale is based upon Rotter’s internal vs. external concept, and higher scores likewise indicate more externality. Maximum scores are not mentioned. Regarding drug regimes, monotherapy was compared with polytherapy. HF patients received more commonly polytherapy and LF patients, monotherapy (*p* < 0.001). Problematic behaviors were more frequently observed in patients with HF and on polytherapy. The LOC scores were 17.4 ± 4.3 for the whole sample, 16.88 ± 4.64 for HF, 15.77 ± 3.66 for LF, and 19.44 ± 4.12 for non-epilepsy controls. The differences were not statistically significant. Thus, LOC seemed to depend more on mental handicap with its restrictions in autonomy than on seizures and antiepileptic drugs.

### Psychogenic Non-epileptic Seizures

The purpose of the Liverpool study of Moore et al. (1994) was to “examine the role of pseudoseizure behavior in fulfilling a function within the family context.” They compared three groups of 19 individuals each with epilepsy, psychogenic non-epileptic seizures (PNES, “pseudoseizures”), and healthy volunteers. Instruments applied were the Family Environment Scale, the HADS, Rosenberg’s self-esteem scale, and for GLOC, the Levenson scales. Of the three LOC scales, only internal LOC was marginally lower in the two seizure groups (13.2 each) than in the control group (14.3), but the difference was not statistically significant.

Stone et al. (2004) compared illness beliefs and LOC of patients with epilepsy and with PNES, both recently diagnosed after a minimum of two seizures, and attending one of two Swedish university hospitals, in a prospective case-control study. For illness beliefs, a Swedish translation of the Illness Behavior Questionnaire was used, and for LOC, a Swedish 50-item self-report instrument. In this, “forty items referred to locus of control orientation with an equal distribution between internal and external directions. The items relate to the concept of locus of control for life events rather than health-specific locus of control. Ten items were adapted from the Karolinska Scales of Personality to measure the degree of social desirability in patients.” In each group, 20 patients were recruited, and there were no healthy controls. Significant differences were that patients with PNES mostly believed in somatic causes of their condition, denied life stresses, and attributed all problems to illness, whereas patients with epilepsy were likely to believe in psychological causes of their condition. The former had a moderately but significantly more external LOC.

Strutt et al. (2011) focused on women with PNES looking for “factors that may potentially aid in the differential diagnosis and subsequent tailoring of treatment.” Thirty patients were diagnosed with PNES and 51 with temporal lobe epilepsy, and areas assessed were motivation, HRQOL, disturbances of mood, and HLOC. Instruments were the Beck depression and anxiety inventories, MMPI, QOLIE-89, and form C of the Wallston et al. scale for HLOC. The PNES patients had a higher score on significant others HLOC (*p* = 0.007).

To conclude, all three studies agree that an increased external or decreased internal LOC in patients with PNES was the only difference from patients with epilepsy to be found.

## Methodological Issues

Investigations of LOC in epilepsy have applied multiple instruments, which make comparisons sometimes difficult. The original one-dimensional internal–external scale of Rotter (1966) is still in use, although it was soon modified by Levenson (1973) into an instrument with three dimensions (internal, chance, and powerful others LOC). This was further developed into a health-related instrument, the W78 with two alternative forms A and B. Wallston et al. (1978) provided normative data for these scales that were based on a chance sample of 115 persons who were encountered at an airport in Nashville, Tennessee, in 1976. Levenson (1973) had a different group of 96 normal controls about whom little is known and which was not referenced later. Their scores, however, were not identical with the Nashville sample, for chance LOC even quite dissimilar. Later, Wallston et al. (1994) developed a form C of W78, which is adaptable to multiple health conditions and has no normative data.

Based upon these traditional instruments, some authors developed adaptations into other languages and cultures or developed their own instruments. The reason may have been that not all items of the original instruments work well in non-United States societies. Thus, Lohse et al. (2015) in Denmark worked with Wallston et al.’s (1994) form C, translated, back-translated, and acknowledged by Wallston. They noted that due to linguistic differences, some patients did not take all questions seriously. Thus, the item “If I am lucky, my epilepsy will get better” in Danish wording sounds like a meaningless commonplace.

For special groups, such as children and individuals with mental handicaps, separate scales were obviously needed and developed.

Numerical values of LOC were given in some studies where they could only be related to normative values from the abovementioned sample of people met at an airport in Nashville, Tennessee, in 1976. Most of the studies were performed so remote from that sample, both in time and space, that these values are not applicable.

Even as the majority of investigators were not interested in absolute values but in correlations of in-group variances of LOC with other parameters, the scarcity of controlled studies is surprising. In fact, only five such studies exist, one of them a small and unrevealing study in mentally handicapped people (Espie et al., 1990). The investigation of Matthews and Barabas (1986) compared small samples of children with epilepsy and diabetes with healthy controls and showed more external LOC in chronically ill children, with a non-significant trend toward more externality in children with epilepsy. Moore et al. (1994) compared three groups (patients with epilepsy, patients with PNES, and healthy controls) and found a non-significant trend toward lower internal LOC in both groups with seizures. The study of Gopinath et al. (2000) in South India compared 200 patients with 206 healthy controls using a locally adapted and validated version of the Rotter (1966) scale. They found a highly significant externalization of LOC in the patients.

In contradistinction, Moritz et al. (2018) in a controlled transcultural study with the Rotter (1966) scale detected no differences in GLOC between patients and controls, and between the two participating countries, Brazil and Lithuania.

As has been repeatedly noted, LOC is a learned concept developing slowly during life and reacting slowly to even important changes of life conditions (Hermann and Wyler, 1989). It is to an important extent dependent on a person’s life situation and ambience. This has been impressively shown by Smith et al. (1995) who created, from 1983 and 1993, a databank of 9,140 responses to the Rotter (1966) scale from employees in business organizations in 43 countries. The country averages ranged widely from 6.35 (Pakistan) to 12.69 (German Democratic Republic). The contrast of the latter with West Germany (8.35) was striking, as the populations of the two (now reunited) countries were in many respects homogeneous but divided by a liberal versus a totalitarian political system. As was discussed earlier (Moritz et al., 2018), the scores were high, expressing external LOC, in almost all of the included highly controlling communist countries (Bulgaria, China, Czechoslovakia, East Germany, Hungary, Poland, Romania, USSR, and Yugoslavia). The point is that “several questions in the scale address success in school and professional life, which in these countries often depended more on compliance with the political system than on one’s abilities.” What was intended to reveal purely subjective perceptions of control turned thus, in these countries, into a description of objective facts with the effect of externalizing the GLOC score. Other relevant aspects beyond individualist vs. collectivist cultures according to Smith et al. (1995) include Christianity vs. religions believing more in fate, but also the demographic composition of nations. Well-matched control groups are, therefore, indispensable whenever generic data about LOC in specific conditions like epilepsy are intended.

Moritz et al. (2018) also proposed another possible factor working toward a “normalization” of LOC scores in epilepsy over time, i.e., reduction of stigmatization of epilepsy due to public awareness campaigns. Repetitive opinion polls measuring public attitudes to epilepsy in several countries such as Czech Republic (Novotná and Rektor, 2017) and United States (Cui et al., 2015) have shown a development toward more inclusion. Unfortunately, studies of the influence of stigma on LOC in epilepsy are largely missing so this remains at present a hypothesis. It is noteworthy, however, that in the same South Indian society, where a significantly externalized LOC was found by Gopinath et al. (2000), public attitudes toward epilepsy at the same time were strongly stigmatizing and excluding (Radhakrishnan et al., 2000).

## Conclusion

The question whether epilepsy has an impact on LOC is not easy to answer on the background of studies with the commonly used instruments. Findings suggesting that epilepsy as such is associated with externalized control perceptions (DeVellis et al., 1980; Matthews and Barabas, 1986) were not methodically robust even if they may have some validity for the United States in the 1980s. They were strongly confirmed for South India by Gopinath et al. (2000) but could, in a transcultural investigation, not be reproduced for countries as far apart as Brazil and Lithuania Moritz et al. (2018). The latter findings seem to indicate that epilepsy as such has no appreciable impact on LOC. Possible accessory reasons for externalization of LOC in epilepsy such as living in a stigmatizing, excluding society have not been sufficiently investigated.

On the other hand, investigations of relative in-group differences of HLOC and GLOC have found some associations. Patients who experience seizure warnings and can meaningfully react to them have a more internal HLOC. Seizure-free patients have a more internal HLOC, and patients with severe epilepsies have a more external HLOC. Patients with a high external HLOC seem to have more difficulties with coping and are perhaps more anxious. Whereas external GLOC was correlated with learned helplessness, internal GLOC was associated with high self-efficacy and better life quality. An association of external LOC with depression seemed not to be a stable co-relation as clinical improvement following epilepsy surgery dissociated the two. Likewise, HLOC was not postoperatively correlated with psychosocial functions. That certain apparent correlations such as external LOC with increased psychopathology and with non-compliance were not confirmed as an independent variable in multiple regression analysis is perhaps not so surprising. LOC seems to be a composite function resulting from multiple factors and as such not a *prima vista* candidate for an independent variable. The correlations may still be interesting.

These findings are not spectacular, and some hypotheses in relation to LOC and epilepsy could also not be confirmed. Thus, the investigations of LOC in epilepsy overall have perhaps not quite lived up to expectations, and there is today less enthusiasm about the topic than there was some decades ago. On the other hand, studies of societal influences like stigmatization and discrimination of epilepsy could provide new interesting data.

## Author Contributions

PW drafted the manuscript and wrote the final version. KL organized the Brazilian arm of the transcultural study on which important conclusions are based, received the invitation to this review and organized it, and edited the draft and approved the final version. RM organized the Lithuanian arm of the transcultural study on which important conclusions are based, and edited the draft and approved the final version. RW made substantial contributions to the draft and approved the final version. All authors contributed to the article and approved the submitted version.

## Conflict of Interest

The authors declare that the research was conducted in the absence of any commercial or financial relationships that could be construed as a potential conflict of interest.
